# Rate-Distortion Region of a Gray–Wyner Model with Side Information

**DOI:** 10.3390/e20010002

**Published:** 2017-12-22

**Authors:** Meryem Benammar, Abdellatif Zaidi

**Affiliations:** 1 Department of Electronics Optronics and Signal Processing (DEOS), Institut Superieur de l’Aéronautique et de l’Espace Supaéro (ISAE Supaéro), 31400 Toulouse, France; 2 Mathematics and Algorithmic Sciences Lab, Huawei Technologies France, 92100 Boulogne-Billancourt, France; abdellatif.zaidi@univ-mlv.fr or

**Keywords:** rate-distortion, Gray–Wyner, side-information, Heegard–Berger, successive refinement

## Abstract

In this work, we establish a full single-letter characterization of the rate-distortion region of an instance of the Gray–Wyner model with side information at the decoders. Specifically, in this model, an encoder observes a pair of memoryless, arbitrarily correlated, sources $\left(S_{1}^{n},S_{2}^{n}\right)$ and communicates with two receivers over an error-free rate-limited link of capacity $R_{0}$, as well as error-free rate-limited individual links of capacities $R_{1}$ to the first receiver and $R_{2}$ to the second receiver. Both receivers reproduce the source component $S_{2}^{n}$ losslessly; and Receiver 1 also reproduces the source component $S_{1}^{n}$ lossily, to within some prescribed fidelity level $D_{1}$. In addition, Receiver 1 and Receiver 2 are equipped, respectively, with memoryless side information sequences $Y_{1}^{n}$ and $Y_{2}^{n}$. Important in this setup, the side information sequences are arbitrarily correlated among them, and with the source pair $\left(S_{1}^{n},S_{2}^{n}\right)$; and are not assumed to exhibit any particular ordering. Furthermore, by specializing the main result to two Heegard–Berger models with *successive refinement* and *scalable coding*, we shed light on the roles of the common and private descriptions that the encoder should produce and the role of each of the common and private links. We develop intuitions by analyzing the developed single-letter rate-distortion regions of these models, and discuss some insightful binary examples.

## 1. Introduction

The Gray–Wyner source coding problem was originally formulated, and solved, by Gray and Wyner in [1]. In their original setting, a pair of arbitrarily correlated memoryless sources $\left(S_{1}^{n},S_{2}^{n}\right)$ is to be encoded and transmitted to two receivers that are connected to the encoder each through a common error-free rate-limited link as well as a private error-free rate-limited link. Because the channels are rate-limited, the encoder produces a compressed bit string $W_{0}$ of rate $R_{0}$ that it transmits over the common link, and two compressed bit strings, $W_{1}$ of rate $R_{1}$ and $W_{2}$ of rate $R_{2}$, that transmits over the private links each to their respective receiver. The first receiver uses the bit strings $W_{0}$ and $W_{1}$ to reproduce an estimate ${\hat{S}}_{1}^{n}$ of the source component $S_{1}^{n}$ to within some prescribed distortion level $D_{1}$, for some distortion measure $d_{1}\left(\cdot ,\cdot \right)$. Similarly, the second receiver uses the bit strings $W_{0}$ and $W_{2}$ to reproduce an estimate ${\hat{S}}_{2}^{n}$ of the source component $S_{2}^{n}$ to within some prescribed distortion level $D_{2}$, for some different distortion measure $d_{2}\left(\cdot ,\cdot \right)$. In [1], Gray and Wyner characterized the optimal achievable rate triples $\left(R_{0},R_{1},R_{2}\right)$ and distortion pairs $\left(D_{1},D_{2}\right)$.

**Figure 1:**
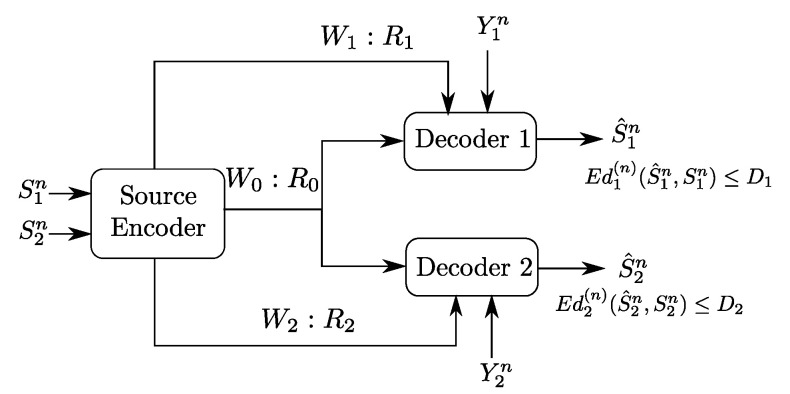
Gray–Wyner network with side information at the receivers.

Figure 1 shows a generalization of the original Gray–Wyner model in which the receivers also observe correlated memoryless side information sequences, $Y_{1}^{n}$ at Receiver 1 and $Y_{2}^{n}$ at Receiver 2. Some special cases of the Gray–Wyner model with side information of Figure 1 have been solved (see the Section 1.2 below). However, in its most general form, i.e., when the side information sequences are arbitrarily correlated among them and with the sources, this problem has so-far eluded single-letter characterization of the optimal rate-distortion region. Indeed, the Gray–Wyner problem with side information subsumes the well known Heegard–Berger problem [2], obtained by setting $R_{1}=R_{2}=0$ in Figure 1, which remains, to date, an open problem.

In this paper, we study an instance of the Gray–Wyner model with side information of Figure 1 in which the reconstruction sets are degraded, meaning, both receivers reproduce the source component $S_{2}^{n}$ losslessly and Receiver 1 wants also to reproduce the source component $S_{1}^{n}$ lossily, to within some prescribed distortion level $D_{1}$. It is important to note that, while the reconstruction sets are nested, and so degraded, no specific ordering is imposed on the side information sequences, which then can be arbitrarily correlated among them and with the sources $\left(S_{1}^{n},S_{2}^{n}\right)$.

As in the Gray–Wyner original coding scheme, the encoder produces a common description of the sources pair $\left(S_{1}^{n},S_{2}^{n}\right)$ that is intended to be recovered by both receivers, as well as individual or private descriptions of $\left(S_{1}^{n},S_{2}^{n}\right)$ that are destined to be recovered each by a distinct receiver. Because the side information sequences do *not* exhibit any specific ordering, the choice of the information that each description should carry, and, the links over which each is transmitted to its intended receiver, are challenging questions that we answer in this work.

**Figure 2:**
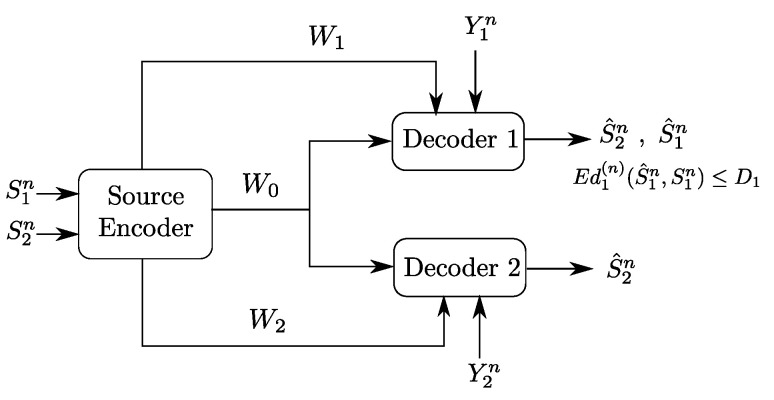
Gray–Wyner model with side information at both receivers and degraded reconstruction sets.

**Figure 3:**
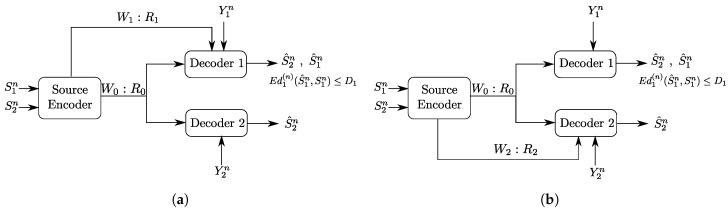
Two classes of Heegard–Berger models (HB models): (**a**) HB model with successive refinement; and (**b**) HB model with scalable coding.

To build the understanding of the role of each of the links and of the descriptions in the optimal coding scheme for the setting of Figure 2, we will investigate as well two important underlying problems, which are Heegard–Berger type models with refinement links as shown in Figure 3. In both models, only one of the two refinement individual links has non-zero rate.

In the model of Figure 3a, the receiver that accesses the additional rate-limited link (i.e., Receiver 1) is also required to reproduce a lossy estimate of the source component $S_{1}^{n}$, in addition to the source component $S_{2}^{n}$ which is to be reproduced losslessly by both receivers. We will refer to this model as a “Heegard–Berger problem with successive refinement”. Reminiscent of successive refinement source coding, this model may be appropriate to model applications in which descriptions of only some components (e.g., $S_{2}^{n}$) of the source suffices at the first use of the data; and descriptions of the remaining components (e.g., $S_{1}^{n}$) are needed only at a later stage.

The model of Figure 3b has the individual rate-limited link connected to the receiver that is required to reproduce only the source component $S_{2}^{n}$. We will refer to this model as a “Heegard–Berger problem with scalable coding”, reusing a term that was introduced in [3] for a similar scenario, and in reference to that user 1 may have such a “good quality” side information that only a minimal amount of information from the encoder suffices, thus, so as not to constrain the communication by user 2 with the lower quality side information, an additional rate limited link $R_{2}$ is added to balance the decoding capabilities of both users.

### 1.1. Main Contributions

The main result of this paper is a single-letter characterization of the optimal rate-distortion region of the Gray–Wyner model with side information and degraded reconstruction sets of Figure 2. To this end, we derive a converse proof that is tailored specifically for the model with degraded reconstruction sets that we study here. For the proof of the direct part, we develop a coding scheme that is very similar to one developed in the context of coding for broadcast channels with feedback in [4], but with an appropriate choice of the variables which we specify here. The specification of the main result to the Heegard–Berger models with successive refinement and scalable coding of Figure 3 sheds light on the roles of the common and private descriptions and what they should carry optimally. We develop intuitions by analyzing the established single-letter optimal rate-distortion regions of these two models, and illustrate our discussion through some binary examples.

### 1.2. Related Works

In [4], Shayevitz and Wigger study a two-receiver discrete memoryless broadcast channel with feedback. They develop an efficient coding scheme which treats the feedback signal as a source that has to be conveyed lossily to the receivers in order to refine their messages’ estimates, through a block Markov coding scheme. In doing so, the users’ channel outputs are regarded as side information sequences; thus, the scheme clearly connects with the Gray–Wyner model with side information of Figure 1—as is also clearly explicit in [4]. The Gray–Wyner model with side information for which Shayevitz and Wigger’s develop a (source) coding scheme, as part of their study of the broadcast channel with feedback, assumes general, possibly distinct, distortion measures at the receivers (i.e., not necessarily nested) and side information sequences that are arbitrarily correlated among them and with the source. In this paper, we show that, when specialized to the model with degraded reconstruction sets of Figure 2 that we study here, Shayevitz and Wigger’s coding scheme for the Gray–Wyner model with side information of [4] yields a rate-distortion region that meets the converse result that we here establish, thus is optimal.

The Gray–Wyner model with side information generalizes another long standing open source coding problem, the famous Heegard–Berger problem [2]. Full single-letter characterization of the optimal rate-distortion function of the Heegard–Berger problem is known only in few specific cases, the most important of which are the cases of : (i) stochastically degraded side information sequences [2] (see also [5]); (ii) Sgarro’s result [6] on the corresponding lossless problem; (iii) Gaussian sources with quadratic distortion measure [3,7]; (iv) some instances of conditionally less-noisy side information sequences [8]; and (v) the recently solved HB model with general side information sequences and degraded reconstruction sets [9], i.e., the model of Figure 2 with $R_{1}=R_{2}=0$— in the lossless case, a few other optimal results were shown, such as for the so-called complementary delivery [10]. A lower bound for general instances of the rate distortion problem with side information at multiple decoders, which is inspired by a linear-programming lower bound for index coding, has been developed recently by Unal and Wagner in [11].

Successive refinement of information was investigated by Equitz et al. in [12], wherein the description of the source is successively refined to a collection of receivers which are required to reconstruct the source with increasing quality levels. Extensions of successive refinement to cases in which the receivers observe some side information sequences was first investigated by Steinberg et al. in [13] who establish the optimal rate-distortion region under the assumption that the receiver that observes the refinement link, say receiver 1, observes also a *better* side information sequence than the opposite user, i.e., the Markov chain S○Y1○Y2 holds. Tian et al. give in [7] an equivalent formulation of the result of [13] and extend it to the N-stage successive refinement setting. In [3], Tian et al. investigate another setting, coined as “side information scalable coding”, in which it is rather the receiver that accesses the refinement link, say receiver 2, which observes the *less good* side information sequence, i.e., S○Y1○Y2. Balancing refinement quality and side information asymmetry for such a side-information scalable source coding problem allows authors in [3] to derive the rate-distortion region in the degraded side information case. The previous results on successive refinement in the presence of side information, which were generalized by Timo et al. in [14], all assume, however, a specific structure in the side information sequences.

### 1.3. Outline

An outline of the remainder of this paper is as follows. Section 2 describes formally the Gray–Wyner model with side information and degraded reconstruction sets of Figure 2 that we study in this paper. Section 3 contains the main result of this paper, a full single-letter characterization of the rate-distortion region of the model of Figure 2, together with some useful discussions and connections. A formal proof of the direct and converse parts of this result appear in Section 6. In Section 4 and Section 5, we specialize the result, respectively, to the Heegard–Berger model with successive refinement of Figure 3a and the Heegard–Berger model with scalable coding of Figure 3b. These sections also contain insightful discussions illustrated by some binary examples.

#### Notation

Throughout the paper, we use the following notations. The term pmf stands for probability mass function. Upper case letters are used to denote random variables, e.g., *X*; lower case letters are used to denote realizations of random variables, e.g., *x*; and calligraphic letters designate alphabets, i.e., X. Vectors of length *n* are denoted by $X^{n}=\left(X_{1},\cdots ,X_{n}\right)$, and $X_{i}^{j}$ is used to denote the sequence $\left(X_{i},\cdots ,X_{j}\right)$, whereas $X_{<i>}≜\left(X_{1},\cdots ,X_{i-1},X_{i+1},\cdots ,X_{n}\right)$. The probability distribution of a random variable *X* is denoted by $P_{X}\left(x\right)≜P\left(X=x\right)$. Sometimes, for convenience, we write it as $P_{X}$. We use the notation E(X) to denote the expectation of a random variable *X*. A probability distribution of a random variable *Y* given *X* is denoted by $P_{Y|X}$. The set of probability distributions defined on an alphabet X is denoted by P(X). The cardinality of a set X is denoted by ∥X∥. For random variables *X*, *Y* and *Z*, the notation X○Y○Z indicates that *X*, *Y* and *Z*, in this order, form a Markov Chain, i.e., $P_{XYZ}\left(x,y,z\right)=P_{Y}\left(y\right)P_{X|Y}\left(x|y\right)P_{Z|Y}\left(z|y\right)$. The set $T_{\left[X\right]}^{\left(n\right)}$ denotes the set of sequences strongly typical with respect to the probability distribution $P_{X}$ and the set $T_{\left[X|y^{n}\right]}^{\left(n\right)}$ denotes the set of sequences $x^{n}$ jointly typical with $y^{n}$ with respect to the joint pmf $P_{XY}$. Throughout this paper, we use $h_{2}\left(\alpha \right)$ to denote the entropy of a Bernoulli (α) random variable, i.e., $h_{2}\left(\alpha \right)=-\alpha \log\left(\alpha \right)-\left(1-\alpha \right)\log\left(1-\alpha \right)$. In addition, the indicator function is denoted by 𝟙(·). For real-valued scalars *a* and *b*, with a≤b, the notation [a,b] means the set of real numbers comprised between *a* and *b*. For integers i≤j, [i:j] denotes the set of integers comprised between *i* and *j*, i.e., [i:j]={i,i+1,⋯,j}. Finally, throughout the paper, logarithms are taken to base 2.

## 2. Problem Setup and Formal Definitions

Consider the Gray–Wyner source coding model with side information and degraded reconstruction sets shown in Figure 2. Let $\left(S_{1}\times S_{2}\times Y_{1}\times Y_{2},P_{S_{1},S_{2},Y_{1},Y_{2}}\right)$ be a discrete memoryless vector source with generic variables $S_{1}$, $S_{2}$, $Y_{1}$ and $Y_{2}$. In addition, let ${\hat{S}}_{1}$ be a reconstruction alphabet and, $d_{1}$ a distortion measure defined as:

(1) $$\begin{array}{ccc} d_{1}\;:\;S_{1}\times {\hat{S}}_{1} & \to & R_{+}\\ \left(s_{1},{\hat{s}}_{1}\right) & \to & d_{1}\left(s_{1},{\hat{s}}_{1}\right)\;. \end{array}$$

**Definition** **1.** *An $\left(n,M_{0,n},M_{1,n},M_{2,n},D_{1}\right)$ code for the Gray–Wyner source coding model with side information and degraded reconstruction sets of Figure 2 consists of:*

- Three sets of messages $W_{0}≜\left[1:M_{0,n}\right]$, $W_{1}≜\left[1:M_{1,n}\right]$, and $W_{2}≜\left[1:M_{2,n}\right]$.
- *Three encoding functions, $f_{0}$, $f_{1}$ and $f_{2}$ defined, for j∈{0,1,2} as*
  (2) $$\begin{array}{ccc} f_{j}\;:\;S_{1}^{n}\times S_{2}^{n} & \mapsto & W_{j}\\ \left(S_{1}^{n},S_{2}^{n}\right) & \mapsto & W_{j}=f_{j}\left(S_{1}^{n},S_{2}^{n}\right)\;. \end{array}$$
- *Two decoding functions $g_{1}$ and $g_{2}$, one at each user:*
  (3) $$\begin{array}{ccc} g_{1}\;:\;W_{0}\times W_{1}\times Y_{1}^{n} & \mapsto & {\hat{S}}_{2}^{n}\times {\hat{S}}_{1}^{n}\\ \left(W_{0},W_{1},Y_{1}^{n}\right) & \mapsto & \left({\hat{S}}_{2,1}^{n},{\hat{S}}_{1}^{n}\right)=g_{1}\left(W_{0},W_{1},Y_{1}^{n}\right)\;, \end{array}$$
  *and*
  (4) $$\begin{array}{ccc} g_{2}\;:\;W_{0}\times W_{2}\times Y_{2}^{n} & \mapsto & {\hat{S}}_{2}^{n}\\ \left(W_{0},W_{2},Y_{2}^{n}\right) & \mapsto & {\hat{S}}_{2,2}^{n}=g_{2}\left(W_{0},W_{2},Y_{2}^{n}\right)\;. \end{array}$$
  *The expected distortion of this code is given by*
  (5) $$E\left(d_{1}^{\left(n\right)}\left(S_{1}^{n},{\hat{S}}_{1}^{n}\right)\right)≜E\frac{1}{n}\sum_{i=1}^{n}d_{1}\left(S_{1,i},{\hat{S}}_{1,i}\right)\;.$$
  *The probability of error is defined as*
  (6) $$P_{e}^{\left(n\right)}≜P\left({\hat{S}}_{2,1}^{n}\neq S_{2}^{n}\;\mathrm{or}\;{\hat{S}}_{2,2}^{n}\neq S_{2}^{n}\right)\;.$$

**Definition** **2.** *A rate triple $\left(R_{0},R_{1},R_{2}\right)$ is said to be $D_{1}$-achievable for the Gray–Wyner source coding model with side information and degraded reconstruction sets of Figure 2 if there exists a sequence of $\left(n,M_{0,n},M_{1,n},M_{2,n},D_{1}\right)$ codes such that:*

(7) $$\begin{array}{ccc} \underset{n\to \infty }{lim sup}P_{e}^{\left(n\right)} & = & 0\;, \end{array}$$

(8) $$\begin{array}{ccc} \underset{n\to \infty }{lim sup}E\left(d_{1}^{\left(n\right)}\left(S_{1}^{n},{\hat{S}}_{1}^{n}\right)\right) & \leq & D_{1}\;, \end{array}$$

(9) $$\begin{array}{ccc} \underset{n\to \infty }{lim sup}\frac{1}{n}\log_{2}\left(M_{j,n}\right) & \leq & R_{j}\;\mathrm{for}\;j\in \left\{0,1,2\right\}. \end{array}$$

*The rate-distortion region RD of this problem is defined as the union of all rate-distortion quadruples $\left(R_{0},R_{1},R_{2},D_{1}\right)$ such that $\left(R_{0},R_{1},R_{2}\right)$ is $D_{1}$-achievable, i.e,*

(10) $$\mathrm{RD}≜\cup \left\{\left(R_{0},R_{1},R_{2},D_{1}\right):\left(R_{0},R_{1},R_{2}\right)\;\mathrm{is}\;D_{1}-\mathrm{achievable}\right\}\;.$$

As we already mentioned, we shall also study the special case Heegard–Berger type models shown in Figure 3. The formal definitions for these models are similar to the above, and we omit them here for brevity.

## 3. Gray–Wyner Model with Side Information and Degraded Reconstruction Sets

In the following, we establish the main result of this work, i.e., the single-letter characterization of the optimal rate-distortion region RD of the Gray–Wyner model with side information and degraded reconstructions sets shown in Figure 2. We then describe how the result subsumes and generalizes existing rate-distortion regions for this setting under different assumptions.

**Theorem** **1.** *The rate-distortion region RD of the Gray–Wyner problem with side information and degraded reconstruction set of Figure 2 is given by the sets of all rate-distortion quadruples $\left(R_{0},R_{1},R_{2},D_{1}\right)$ satisfying:*

(11a) $$\begin{array}{ccc} R_{0}+R_{1} & \geq & H\left(S_{2}|Y_{1}\right)+I\left(U_{0}U_{1};S_{1}|S_{2}Y_{1}\right) \end{array}$$

(11b) $$\begin{array}{ccc} R_{0}+R_{2} & \geq & H\left(S_{2}|Y_{2}\right)+I\left(U_{0};S_{1}|S_{2}Y_{2}\right) \end{array}$$

(11c) $$\begin{array}{ccc} R_{0}+R_{1}+R_{2} & \geq & H\left(S_{2}|Y_{2}\right)+I\left(U_{0};S_{1}|S_{2}Y_{2}\right)+I\left(U_{1};S_{1}|U_{0}S_{2}Y_{1}\right) \end{array}$$

*for some product pmf $P_{U_{0}U_{1}S_{1}S_{2}Y_{1}Y_{2}}$, such that:*

*(1)*  *The following Markov chain is valid:*
  (12) (Y1,Y2)○(S1,S2)○(U0,U1)
*(2)*  *There exists a function $\phi :Y_{1}\times U_{0}\times U_{1}\times S_{2}\to {\hat{S}}_{1}$ such that:*
  (13) $$Ed_{1}\left(S_{1},{\hat{S}}_{1}\right)\leq D_{1}\;.$$

**Proof.**  The detailed proof of the direct part and the converse part of this theorem appear in Section 6.

The proof of converse, which is the most challenging part, uses appropriate combinations of bounding techniques for the transmitted rates based on the system model assumptions and Fano’s inequality, a series of analytic bounds based on the underlying Markov chains, and most importantly, a proper use of Csiszár–Körner sum identity in order to derive single letter bounds.

As for the proof of achievability, it combines the optimal coding scheme of the Heegard–Berger problem with degraded reconstruction sets [9] and the double-binning based scheme of Shayevitz and Wigger (Theorem 2, [4]) for the Gray–Wyner problem with side information, and is outlined in the following.

The encoder produces a common description of $\left(S_{1}^{n},S_{2}^{n}\right)$ that is intended to be recovered by both receivers, and an individual description that is intended to be recovered only by Receiver 1. The common description is chosen as $V_{0}^{n}=\left(U_{0}^{n},S_{2}^{n}\right)$ and is thus designed to describe all of $S_{2}^{n}$, which both receivers are required to reproduce lossessly, but also all or part of $S_{1}^{n}$, depending on the desired distortion level $D_{1}$. Since we make no assumptions on the side information sequences, this is meant to account for possibly unbalanced side information pairs $\left(Y_{1}^{n},Y_{2}^{n}\right)$, in a manner that is similar to [9]. The message that carries the common description is obtained at the encoder through the technique of double-binning of Tian and Diggavi in [3], used also by Shayevitz and Wigger (Theorem 2, [4]) for a Gray–Wyner model with side information. In particular, similar to the coding scheme of (Theorem 2, [4]), the double-binning is performed in two ways, one that is tailored for Receiver 1 and one that is tailored for Receiver 2.

More specifically, the codebook of the common description is composed of codewords $v_{0}^{n}$ that are drawn randomly and independently according to the product law of $P_{V_{0}}$; and is partitioned uniformly into $2^{n{\tilde{R}}_{0,0}}$ superbins, indexed with ${\tilde{w}}_{0,0}\in \left[1:2^{n{\tilde{R}}_{0,0}}\right]$. The codewords of each superbin of this codebook are partitioned in two distinct ways. In the first partition, they are assigned randomly and independently to $2^{n{\tilde{R}}_{0,1}}$ subbins indexed with ${\tilde{w}}_{0,1}\in \left[1:2^{n{\tilde{R}}_{0,1}}\right]$, according to a uniform pmf over $\left[1:2^{n{\tilde{R}}_{0,1}}\right]$. Similarly, in the second partition, they are assigned randomly and independently to $2^{n{\tilde{R}}_{0,2}}$ subbins indexed with ${\tilde{w}}_{0,2}\in \left[1:2^{n{\tilde{R}}_{0,2}}\right]$, according to a uniform pmf over $\left[1:2^{n{\tilde{R}}_{0,2}}\right]$. The codebook of the private description is composed of codewords $u_{1}^{n}$ that are drawn randomly and independently according to the product law of $P_{U_{1}|V_{0}}$. This codebook is partitioned similarly uniformly into $2^{n{\tilde{R}}_{1,0}}$ superbins indexed with ${\tilde{w}}_{1,0}\in \left[1:2^{n{\tilde{R}}_{1,0}}\right]$, each containing $2^{n{\tilde{R}}_{1,1}}$ subbins indexed with ${\tilde{w}}_{1,1}\in \left[1:2^{n{\tilde{R}}_{1,1}}\right]$ codewords $u_{1}^{n}$.

Upon observing a typical pair $\left(S_{1}^{n},S_{2}^{n}\right)=\left(s_{1}^{n},s_{2}^{n}\right)$, the encoder finds a pair of codewords $\left(v_{0}^{n},u_{1}^{n}\right)$ that is jointly typical with $\left(s_{1}^{n},s_{2}^{n}\right)$. Let ${\tilde{w}}_{0,0}$, ${\tilde{w}}_{0,1}$ and ${\tilde{w}}_{0,2}$ denote respectively the indices of the superbin, subbin of the first partition and subbin of the second partition of the codebook of the common description, in which lies the found $v_{0}^{n}$. Similarly, let ${\tilde{w}}_{1,0}$ and ${\tilde{w}}_{1,1}$ denote respectively the indices of the superbin and subbin of the codebook of the individual description in which lies the found $u_{1}^{n}$. The encoder sets the common message $W_{0}$ as $W_{0}=\left({\tilde{w}}_{0,0},{\tilde{w}}_{1,0}\right)$ and sends it over the error-free rate-limited common link of capacity $R_{0}$. In addition, it sets the individual message $W_{1}$ as $W_{1}=\left({\tilde{w}}_{0,1},{\tilde{w}}_{1,1}\right)$ and sends it the error-free rate-limited link to Receiver 1 of capacity $R_{1}$; and the individual message $W_{2}$ as $W_{2}={\tilde{w}}_{0,2}$ and sends it the error-free rate-limited link to Receiver 2 of capacity $R_{2}$. For the decoding, Receiver 2 utilizes the second partition of the codebook of the common description; and looks in the subbin of index ${\tilde{w}}_{0,2}$ of the superbin of index ${\tilde{w}}_{0,0}$ for a unique $v_{0}^{n}$ that is jointly typical with its side information $y_{2}^{n}$. Receiver 1 decodes $v_{0}^{n}$ similarly, utilizing the first partition of the codebook of the common description and its side information $y_{1}^{n}$. It also utilizes the codebook of the individual description; and looks in the subbin of index ${\tilde{w}}_{1,1}$ of the superbin of index ${\tilde{w}}_{1,1}$ for a unique $u_{1}^{n}$ that is jointly typical with the pair $\left(y_{1}^{n},v_{0}^{n}\right)$. In the formal proof in Section IV, we argue that with an appropropriate choice of the communication rates ${\tilde{R}}_{0,0}$, ${\tilde{R}}_{0,1}$, ${\tilde{R}}_{0,2}$, ${\tilde{R}}_{1,0}$ and ${\tilde{R}}_{1,1}$, as well as the sizes of the subbins, this scheme achieves the rate-distortion region of Theorem 1. ☐

A few remarks that connect Theorem 1 to known results on related models are in order.

**Figure 4:**
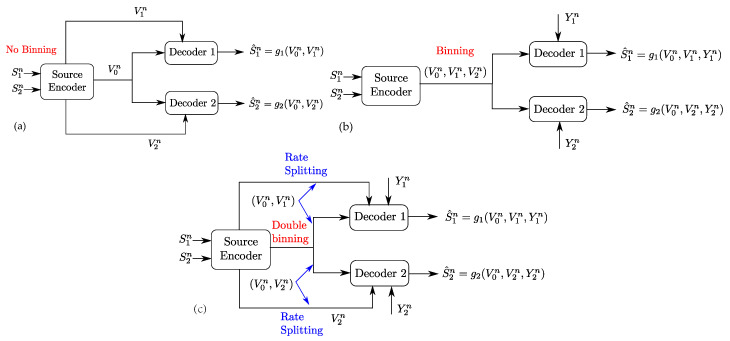
Comparison of coding schemes for the Gray–Wyner network with side information, Gray–Wyner network and the Heegard–Berger problem: (**a**) coding scheme for the Gray–Wyner network; (**b**) coding scheme for the Heegard–Berger problem; and (**c**) coding scheme for the Gray–Wyner network with side information.

**Remark** **1.** The setting of Figure 1 generalizes two important settings which are the Gray–Wyner problem, through the presence of side-information sequences $Y_{1}^{n}$ and $Y_{2}^{n}$, and the Heegard–Berger problem, through the presence of private links of rates $R_{1}$ and $R_{2}$. As such, the coding scheme for the setting of Figure 2 differs from that of the Gray–Wyner problem and that of the Heegard–Berger problem in many aspects as shown in Figure 4.

First, the presence of side information sequences imposes the use of “binning” for each of the produced descriptions $V_{0}^{n},V_{1}^{n}$ and $V_{2}^{n}$ in the Gray–Wyner code construction. However, unlike the binning performed in the Heegard–Berger coding scheme, the binning of the common codeword $V_{0}^{n}$ needs to be performed with two different indices, each tailored to a side information sequence at the respective receivers, i.e., “double binning”. Another different aspect is the role of the private and common links. When in the original Gray–Wyner work, these links carried each a description, i.e., $V_{0}^{n}$ on the common link and $V_{1}^{n}$ with respect to $V_{2}^{n}$ on the private links of rates $R_{1}$ with respect to $R_{2}$, and when in the Heegard–Berger the three descriptions $V_{0}^{n},V_{1}^{n}$ and $V_{2}^{n}$ are all carried through the common link only, in the optimal coding scheme of the setting of Figure 2, the private and common links play different roles. Indeed, the common description $V_{0}^{n}$ and the private description $V_{j}^{n}$ are transmitted on both the common link and the private link of rates $R_{0}$ and $R_{j}$, for j∈{1,2}, through rate-splitting. As such, these key differences imply an intricate interplay between the side information sequences and the role of the common and private links, which we will emphasize later on in Section 4 and Section 5.

**Remark** **2.** In the special case in which $R_{1}=R_{2}=0$, the Gray–Wyner model with side information and degraded reconstruction sets of Figure 2 reduces to a Heegard–Berger problem with arbitrary side information sequences and degraded reconstruction sets, a model that was studied, and solved, recently in the authors’ own recent work [9]. Theorem 1 can then be seen as a generalization of (Theorem 1, [9]) to the case in which the encoder is connected to the receivers also through error-free rate-limited private links of capacity $R_{1}$ and $R_{2}$ respectively. The most important insight in the Heegard–Berger problem with degraded reconstruction sets is the role that the common description $V_{0}$ should play in such a setting. Authors show in (Theorem 1, [9]) that the optimal choice of this description is to contain, intuitively, the common source $S_{2}$ intended to both users, and, maybe less intuitive, an additional description $U_{0}$, i.e., $V_{0}=\left(U_{0},S_{2}\right)$, which is used to piggyback part of the source $S_{1}$ in the common codeword though not required by both receivers, in order to balance the asymmetry of the side information sequences. In Section 4 and Section 5 we show that the utility of this description will depend on both the side information sequences and the rates of the private links.

**Remark** **3.** In [15], Timo et al. study the Gray–Wyner source coding model with side information of Figure 1. They establish the rate-region of this model in the specific case in which the side information sequence $Y_{2}^{n}$ is a degraded version of $Y_{1}^{n}$, i.e., (S1,S2)○Y1○Y2 is a Markov chain, and both receivers reproduce the component $S_{2}^{n}$ and Receiver 1 also reproduces the component $S_{1}^{n}$, all in a lossless manner. The result of Theorem 1 generalizes that of (Theorem 5, [15]) to the case of side information sequences that are arbitrarily correlated among them and with the source pair $\left(S_{1},S_{2}\right)$ and lossy reconstruction of $S_{1}$. In [15], Timo et al. also investigate, and solve, a few other special cases of the model, such as those of single source $S_{1}=S_{2}$ (Theorem 4, [15]) and complementary delivery $\left(Y_{1},Y_{2}\right)=\left(S_{2},S_{1}\right)$ (Theorem 6, [15]). The results of (Theorem 4, [15]) and (Theorem 6, [15]) can be recovered from Theorem 1 as special cases of it. Theorem 1 also generalizes (Theorem 6, [15]) to the case of lossy reproduction of the component $S_{1}^{n}$.

## 4. The Heegard–Berger Problem with Successive Refinement

An important special case of the Gray–Wyner source coding model with side information and degraded reconstruction sets of Figure 2 is the case in which $R_{2}=0$. The resulting model, a Heegard–Berger problem with successive refinement, is shown in Figure 3a.

In this section, we derive the optimal rate distortion region for this setting, and show how it compares to existing results in literature. Besides, we discuss the utility of the common description $U_{0}$ depending, not only on the side information sequences structures, but also on the refinement link rate $R_{1}$. We illustrate through a binary example that the utility of $U_{0}$, namely the optimality of the choice of a non-degenerate $U_{0}\neq ⌀$, is governed by the quality of the refinement link rate $R_{1}$ and the side information structure.

### 4.1. Rate-Distortion Region

The following theorem states the optimal rate-distortion region of the Heegard–Berger problem with successive refinement of Figure 3a.

**Corollary** **1.** *The rate-distortion region of the Heegard–Berger problem with successive refinement of Figure 3a is given by the set of rate-distortion triples $\left(R_{0},R_{1},D_{1}\right)$ satisfying:*

(14a) $$\begin{array}{ccc} R_{0} & \geq & H\left(S_{2}|Y_{2}\right)+I\left(U_{0};S_{1}|S_{2}Y_{2}\right) \end{array}$$

(14b) $$\begin{array}{ccc} R_{0}+R_{1} & \geq & H\left(S_{2}|Y_{1}\right)+I\left(U_{0}U_{1};S_{1}|S_{2}Y_{1}\right) \end{array}$$

(14c) $$\begin{array}{ccc} R_{0}+R_{1} & \geq & H\left(S_{2}|Y_{2}\right)+I\left(U_{0};S_{1}|S_{2}Y_{2}\right)+I\left(U_{1};S_{1}|U_{0}S_{2}Y_{1}\right) \end{array}$$

*for some product pmf $P_{U_{0}U_{1}S_{1}S_{2}Y_{1}Y_{2}}$, such that:*

*(1)*  *The following Markov chain is valid:*
  (15) (U0,U1)○(S1,S2)○(Y1,Y2)

*(2)*  *There exists a function $\phi :Y_{1}\times U_{0}\times U_{1}\times S_{2}\to {\hat{S}}_{1}$ such that:*
  (16) $$Ed_{1}\left(S_{1},{\hat{S}}_{1}\right)\leq D_{1}\;.$$

**Proof.**  The proof of Corollary 1 follows from that of Theorem 1 by setting $R_{2}=0$ therein. ☐

**Remark** **4.** *Recall the coding scheme of Theorem 1. If $R_{2}=0$, the second partition of the codebook of the common description, which is relevant for Receiver 2, becomes degenerate since, in this case, all the codewords $v_{0}^{n}$ of a superbin $B_{00}\left({\tilde{w}}_{0,0}\right)$ are assigned to a single subbin. Correspondingly, the common message that the encoder sends over the common link carries only the index ${\tilde{w}}_{0,0}$ of the superbin $B_{00}\left({\tilde{w}}_{0,0}\right)$ of the codebook of the common description in which lies the typical pair $v_{0}^{n}=\left(s_{2}^{n},u_{0}^{n}\right)$, in addition to the index ${\tilde{w}}_{1,0}$ of the subbin $B_{10}\left({\tilde{w}}_{1,0}\right)$ of the codebook of the individual description in which lies the recovered typical $u_{1}^{n}$. Constraint (14a) on the common rate $R_{0}$ is in accordance with that Receiver 2 utilizes only the index ${\tilde{w}}_{0,0}$ in the decoding. Furthermore, note that Constraints (14b) and (14c) on the sum-rate $\left(R_{0}+R_{1}\right)$ can be combined as*

(17) $$R_{0}+R_{1}\geq \max\;\left\{I\left(U_{0}S_{2};S_{1}S_{2}|Y_{1}\right),I\left(U_{0}S_{2};S_{1}S_{2}|Y_{2}\right)\right\}+I\left(U_{1};S_{1}|U_{0}S_{2}Y_{1}\right)$$

*which resembles the Heegard–Berger result of (Theorem 2, p. 733, [2]).*

**Remark** **5.** *As we already mentioned, the result of Corollary 1 holds for side information sequences that are arbitrarily correlated among them and with the sources. In the specific case in which the user who gets the refinement rate-limited link also has the “better-quality” side information, in the sense that (S1,S2)○Y1○Y2 forms a Markov chain, the rate-distortion region of Corollary 1 reduces to the set of all rate-distortion triples $\left(R_{0},R_{1},D_{1}\right)$ that satisfy*

(18a) $$\begin{array}{ccc} R_{0} & \geq & H\left(S_{2}|Y_{2}\right)+I\left(U_{0};S_{1}|S_{2}Y_{2}\right) \end{array}$$

(18b) $$\begin{array}{ccc} R_{0}+R_{1} & \geq & H\left(S_{2}|Y_{2}\right)+I\left(U_{0};S_{1}|S_{2}Y_{2}\right)+I\left(U_{1};S_{1}|U_{0}S_{2}Y_{1}\right)\;. \end{array}$$

*for some joint pmf $P_{U_{0}U_{1}S_{1}S_{2}Y_{1}Y_{2}}$ for which (15) and (16) hold. This result can also be obtained from previous works on successive refinement for the Wyner–Ziv source coding problem by Steinberg and Merhav (Theorem 1, [13]) and Tian and Diggavi (Theorem 1, [7]). The results of (Theorem 1, [13]) and (Theorem 1, [7]) hold for possibly distinct, i.e., not necessarily nested, distortion measures at the receivers; but they require the aforementioned Markov chain condition which is pivotal for their proofs. Thus, for the considered degraded reconstruction sets setting, Corollary 1 can be seen as generalizing (Theorem 1, [13]) and (Theorem 1, [7]) to the case in which the side information sequences are arbitrarily correlated among them and with the sources $\left(S_{1},S_{2}\right)$, i.e., do not exhibit any ordering.*

**Remark** **6.** *In the case in which it is the user who gets only the common rate-limited link that has the “better-quality” side information, in the sense that (S1,S2)○Y2○Y1 forms a Markov chain, the rate distortion region of Corollary 1 reduces to the set of all rate-distortion triples $\left(R_{0},R_{1},D_{1}\right)$ that satisfy*

(19a) $$\begin{array}{ccc} R_{0} & \geq & H\left(S_{2}|Y_{2}\right)+I\left(U_{0};S_{1}|S_{2}Y_{2}\right) \end{array}$$

(19b) $$\begin{array}{ccc} R_{0}+R_{1} & \geq & H\left(S_{2}|Y_{1}\right)+I\left(U_{0}U_{1};S_{1}|S_{2}Y_{1}\right) \end{array}$$

*for some joint pmf $P_{U_{0}U_{1}S_{1}S_{2}Y_{1}Y_{2}}$ for which (15) and (16) hold. This result can also be conveyed from [3]. Specifically, in [3] Tian and Diggavi study a therein referred to as “side-information scalable” source coding setup where the side informations are degraded, and the encoder produces two descriptions such that the receiver with the better-quality side information (Receiver 2 if (S1,S2)○Y2○Y1 is a Markov chain) uses only the first description to reconstruct its source while the receiver with the low-quality side information (Receiver 1 if (S1,S2)○Y2○Y1 is a Markov chain) uses the two descriptions in order to reconstruct its source. They establish inner and outer bounds on the rate-distortion region of the model, which coincide when either one of the decoders requires a lossless reconstruction or when the distortion measures are degraded and deterministic. Similar to the previous remark, Corollary 1 can be seen as generalizing the aforementioned results of [3] to the case in which the side information sequences are arbitrarily correlated among them and with the sources $\left(S_{1},S_{2}\right)$.*

**Remark** **7.** A crucial remark that is in order for the Heegard–Berger problem with successive refinement of Figure 3a, is that, depending on the rate of the refinement link $R_{1}$, resorting to a common auxiliary variable $U_{0}$ might be unnecessary. Indeed, in the case in which $S_{1}$ needs to be recovered losslessly at the first receiver, for instance, parts of the rate-region can be achieved without resorting to the common auxiliary variable $U_{0}$, setting $U_{0}=⌀$, while other parts of the rate region can only be achieved through a non-trivial choice of $U_{0}$.

*As such, if $R_{1}\geq H\left(S_{1}|S_{2}Y_{1}\right)$, then letting $U_{0}=⌀$ yields the optimal rate region. To see this, note that the rate constraints under lossless construction of $S_{1}$ write as:*

(20a) $$\begin{array}{ccc} R_{0} & \geq & H\left(S_{1}S_{2}|Y_{2}\right)-H\left(S_{1}|S_{2}Y_{2}U_{0}\right) \end{array}$$

(20b) $$\begin{array}{ccc} R_{0}+R_{1} & \geq & H(S_{1}S_{2}|Y_{1}) \end{array}$$

(20c) $$\begin{array}{ccc} R_{0}+R_{1} & \geq & H\left(S_{1}S_{2}|Y_{2}\right)-H\left(S_{1}|S_{2}Y_{2}U_{0}\right)+H\left(S_{1}|U_{0}S_{2}Y_{1}\right) \end{array}$$

*which, can be rewritten as follows*

(21a) $$\begin{array}{ccc} {}*R_{0} & \geq & H\left(S_{1}S_{2}|Y_{2}\right)+\min_{P_{U_{0}|S_{1}S_{2}}}\left[{\left(H\left(S_{1}|S_{2}Y_{1}U_{0}\right)-R_{1}\right)}^{+}-H\left(S_{1}|S_{2}Y_{2}U_{0}\right)\right] \end{array}$$

(21b) $$\begin{array}{ccc} R_{0}+R_{1} & \geq & H(S_{1}S_{2}|Y_{1}) \end{array}$$

*where ${\left(x\right)}^{+}≜\max\left\{0,x\right\}$.*

*Under the constraint that $R_{1}\geq H\left(S_{1}|S_{2}Y_{1}\right)$, the constraints in (21a) reduce to the following*

(22a) $$\begin{array}{ccc} R_{0} & \geq & H\left(S_{1}S_{2}|Y_{2}\right)-\max_{P_{U_{0}|S_{1}S_{2}}}H\left(S_{1}|S_{2}Y_{2}U_{0}\right) \end{array}$$

(22b) $$\begin{array}{ccc} R_{0}+R_{1} & \geq & H(S_{1}S_{2}|Y_{1}). \end{array}$$

Next, by noting that $\max_{P_{U_{0}|S_{1}S_{2}}}H\left(S_{1}|S_{2}Y_{2}U_{0}\right)=H\left(S_{1}|S_{2}Y_{2}\right)$ is achieved by $U_{0}=⌀$, the claim follows.

However, when $R_{1}<H\left(S_{1}|S_{2}Y_{1}\right)$, the choice of $U_{0}=⌀$ might be strictly sub-optimal (as shown in the following binary example).

### 4.2. Binary Example

**Figure 5:**
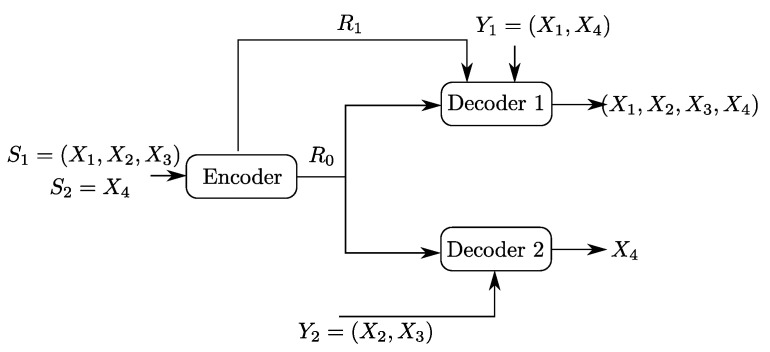
Binary Heegard–Berger example with successive refinement.

Let $X_{1}$, $X_{2}$, $X_{3}$ and $X_{4}$ be four independent Ber(1/2) random variables. Let the sources be $S_{1}≜\left(X_{1},X_{2},X_{3}\right)$ and $S_{2}≜X_{4}$. Now, consider the Heegard–Berger model with successive refinement shown in Figure 5. The first user, which gets both the common and individual links, observes the side information $Y_{1}=\left(X_{1},X_{4}\right)$ and wants to reproduce the pair $\left(S_{1},S_{2}\right)$ losslessly. The second user gets only the common link, has side information $Y_{2}=\left(X_{2},X_{3}\right)$ and wants to reproduce only the component $S_{2}$, losslessly.

The side information at the decoders do *not* exhibit any degradedness ordering, in the sense that none of the Markov chain conditions of Remarks 5 and 6 hold. The following claim provides the rate-region of this binary example.

**Claim** **1.** *The rate region of the binary Heegard–Berger example with successive refinement of Figure 5 is given by the set of rate pairs $\left(R_{0},R_{1}\right)$ that satisfy*

(23a) $$\begin{array}{ccc} R_{0} & \geq & 1 \end{array}$$

(23b) $$\begin{array}{ccc} R_{0}+R_{1} & \geq & 2\;. \end{array}$$

**Proof.**  The proof of Claim 1 follows easily by computing the rate region

(24a) $$\begin{array}{ccc} R_{0} & \geq & H\left(S_{1}S_{2}|Y_{2}\right)-H\left(S_{1}|S_{2}Y_{2}U_{0}\right) \end{array}$$

(24b) $$\begin{array}{ccc} R_{0}+R_{1} & \geq & H(S_{1}S_{2}|Y_{1}) \end{array}$$

(24c) $$\begin{array}{ccc} R_{0}+R_{1} & \geq & H\left(S_{1}S_{2}|Y_{2}\right)-H\left(S_{1}|S_{2}Y_{2}U_{0}\right)+H\left(S_{1}|U_{0}S_{2}Y_{1}\right) \end{array}$$

in the binary setting under study.

First, we note that

(25) $$\begin{array}{ccc} H(S_{1}S_{2}|Y_{2}) & = & H(X_{1}X_{4}|X_{2}X_{3})=2 \end{array}$$

(26) $$\begin{array}{ccc} H(S_{1}S_{2}|Y_{1}) & = & H(X_{2}X_{3}|X_{1}X_{4})=2 \end{array}$$

which allows then to rewrite the rate region as

(27a) $$\begin{array}{cc} R_{0} & \geq 2-H\left(X_{1}|X_{4}U_{0}\right)\geq 2-H\left(X_{1}|X_{4}\right)=1 \end{array}$$

(27b) $$\begin{array}{cc} R_{0}+R_{1} & \geq 2+\max\{0,H\left(X_{2}X_{3}|X_{1}X_{4}U_{0}\right)-H\left(X_{1}|X_{2}X_{3}X_{4}U_{0}\right)\}\geq 2 \end{array}$$

The proof of the claim follows by noticing that the following inequalities hold with equality for the choices $U_{0}=\left(X_{2},X_{3}\right)$ or $U_{0}=X_{2}$ or $U_{0}=X_{3}$. ☐

**Figure 6:**
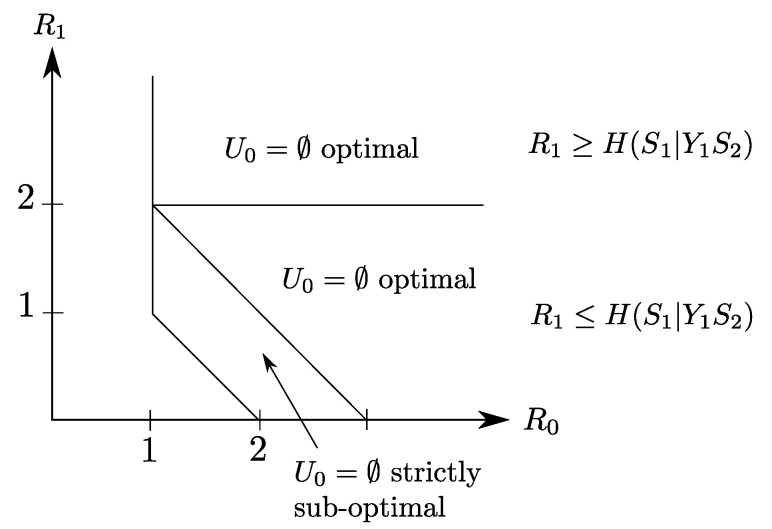
Rate region of the binary example of Figure 5. The choices $U_{0}=\left(X2,X3\right)$ or $U_{0}=X_{2}$ or $U_{0}=X_{3}$ are optimal irrespective of the value of $R_{1}$, while the degenerate choice $U_{0}=⌀$ is optimal only in some slices of the region.

The rate region of Claim 1 is depicted in Figure 6. It is insightful to notice that although the second user is only interested in reproducing the component $S_{2}=X_{4}$, the optimal coding scheme that achieves this region sets the common description that is destined to be recovered by both users as one that is composed of not only $S_{2}$ but also some part $U_{0}=\left(X_{2},X_{3}\right)$, or $U_{0}=X_{2}$ or $U_{0}=X_{3}$, of the source component $S_{1}$ (though the latter is not required by the second user). A possible intuition is that this choice of $U_{0}$ is useful for user 1, who wants to reproduce $S_{1}=\left(X_{1},X_{2},X_{3}\right)$, and its transmission to also the second user does not cost any rate loss since this user has available side information $Y_{2}=\left(X_{2},X_{3}\right)$.

## 5. The Heegard–Berger Problem with Scalable Coding

In the following, we consider the model of Figure 3b. As we already mentioned, the reader may find it appropriate for the motivation to think about the side information $Y_{2}^{n}$ as being of lower quality than $Y_{1}^{n}$, in which case, the refinement link that is given to the second user is intended to improve its decoding capability. In this section, we describe the optimal coding scheme for this setting, and show that it can be recovered, independently, from the work of Timo et al. [14] through a careful choice of the coding sets. Next, we illustrate through a binary example the interplay between the utility of the common description $U_{0}$ and the side information sequences, and the refinement rate $R_{2}$.

### 5.1. Rate-Distortion Region

The following theorem states the rate-distortion region of the Heegard–Berger model with scalable coding of Figure 3b.

**Corollary** **2.** *The rate-distortion region of the Heegard–Berger model with scalable coding of Figure 3b is given by the set of all rate-distortion triples $\left(R_{0},R_{2},D_{1}\right)$ that satisfy*

(28a) $$\begin{array}{ccc} R_{0} & \geq & H\left(S_{2}|Y_{1}\right)+I\left(U_{0}U_{1};S_{1}|S_{2}Y_{1}\right) \end{array}$$

(28b) $$\begin{array}{ccc} R_{0}+R_{2} & \geq & H\left(S_{2}|Y_{2}\right)+I\left(U_{0};S_{1}|S_{2}Y_{2}\right)+I\left(U_{1};S_{1}|U_{0}S_{2}Y_{1}\right) \end{array}$$

*for some product pmf $P_{U_{0}U_{1}S_{1}S_{2}Y_{1}Y_{2}}$, such that:*

*(1)*  *The following Markov chain is valid:*
  (29) (U0,U1)○(S1,S2)○(Y1,Y2)
*(2)*  *There exists a function $\phi :Y_{1}\times U_{0}\times U_{1}\times S_{2}\to {\hat{S}}_{1}$ such that:*
  (30) $$Ed_{1}\left(S_{1},{\hat{S}}_{1}\right)\leq D_{1}\;.$$

**Proof.**  The proof of Corollary 2 follows from that of Theorem 1 by seeting $R_{1}=0$ therein. ☐

**Remark** **8.** *In the specific case in which Receiver 2 has a better-quality side information in the sense that (S1,S2)○Y2○Y1 forms a Markov chain, the rate distortion region of Corollary 2 reduces to one that is described by a single rate-constraint, namely*

(31) $$\begin{array}{ccc} R_{0} & \geq & H\left(S_{2}|Y_{1}\right)+I\left(U;S_{1}|S_{2}Y_{1}\right) \end{array}$$

*for some conditional $P_{U|S_{1}S_{2}}$ that satisfies $E\left[d_{1}\left(S_{1},{\hat{S}}_{1}\right)\right]\leq D_{1}$. This is in accordance with the observation that, in this case, the transmission to Receiver 1 becomes the bottleneck, as Receiver 2 can recover the source component $S_{2}$ losslessly as long as so does Receiver 1.*

**Remark** **9.** *Consider the case in which $S_{1}$ needs to be recovered losslessly as well at Receiver 1. Then, the rate region is can be expressed as follows*

(32a) $$\begin{array}{cc} R_{0} & \geq H(S_{1}S_{2}|Y_{1}) \end{array}$$

(32b) $$\begin{array}{cc} R_{0}+R_{2} & \geq H\left(S_{1}S_{2}|Y_{2}\right)+\min_{P_{U_{0}|S_{1}S_{2}}}\left[H\left(S_{1}|U_{0}S_{2}Y_{1}\right)-H\left(S_{1}|U_{0}S_{2}Y_{2}\right)\right]. \end{array}$$

An important comment here is that the optimization problem in $P_{U_{0}|S_{1}S_{2}}$ does not depend on the refinement link $R_{2}$, and the optimal solution to it, i.e., the optimal choice of $U_{0}$, meets the solution to the Heegard–Berger problem without refinement link, $R_{2}=0$, rendering it optimal for all choices of $R_{2}$, which is a main difference with the Heegard–Berger problem with refinement link of Figure 3a in which the solution to the Heegard–Berger problem (with $R_{1}=0$) might not be optimal for all values of $R_{1}$.

**Remark** **10.** *In (Theorem 1, [14]), Timo et al. present an achievable rate-region for the multistage successive-refinement problem with side information. Timo et al. consider distortion measures of the form $\delta_{l}\;:\;X\times {\hat{X}}_{l}\to R_{+}$, where X is the source alphabet and ${\hat{X}}_{l}$ is the reconstruction at decoder l, l∈{1,⋯,t}; and for this reason this result is not applicable as is to the setting of Figure 3b, in the case of two decoders. However, the result of (Theorem 1, [14]) can be extended to accommodate a distortion measure at the first decoder that is vector-valued; and the direct part of Corollary 2 can then be obtained by applying this extension. Specifically, in the case of two decoders, i.e., t=2, and with $X=(S_{1},S_{2})$, and two distortion measures $\delta_{1}:S_{1}\times S_{2}\times {\hat{S}}_{1,1}\times {\hat{S}}_{1,2}\to \left\{0,1\right\}\times R_{+}$ and $\delta_{2}:S_{1}\times S_{2}\times {\hat{S}}_{1,2}\times {\hat{S}}_{2,2}\to \left\{0,1\right\}$ chosen such that*

(33) $$\delta_{1}\left(\left(s_{1},s_{2}\right),\left({\hat{s}}_{1,1},{\hat{s}}_{2,1}\right)\right)=\left(d_{H}\left(s_{2},{\hat{s}}_{2,1}\right),d_{1}\left(s_{1},{\hat{s}}_{1,1}\right)\right)$$

*and*

(34) $$\delta_{2}\left(\left(s_{1},s_{2}\right),\left({\hat{s}}_{1,2},{\hat{s}}_{2,2}\right)\right)=d_{H}\left(s_{2},{\hat{s}}_{2,2}\right)$$

*where $d_{H}\left(\cdot ,\cdot \right)$ is the Hamming distance, letting $d_{1}=\left(0,D_{1}\right)$ and $d_{2}=0$, a straightforward extension of (Theorem 1, [14]) to this setting yields a rate-region that is described by the following rate constraints (using the notation of (Theorem 1, [14]))*

(35a) $$\begin{array}{cc} R_{0} & \geq \Phi \left(T_{0},1\right)+\Phi \left(T_{1},1\right) \end{array}$$

(35b) $$\begin{array}{cc} R_{0}+R_{2} & \geq \Phi \left(T_{0},2\right)+\Phi \left(T_{1},2\right)+\Phi \left(T_{2},2\right) \end{array}$$

*where $T_{0}=\left\{1,2\right\}$, $T_{1}=\left\{1\right\}$, $T_{2}=\left\{2\right\}$, and for j=0,1,2 and l∈1,2 such that $T_{j}\cap \left\{1,\cdots ,l\right\}\neq ⌀$, the function $\Phi (T_{j},l)$, j=0,1,2, is defined as*

(36) $$\Phi \left(T_{j},l\right)=I\left(S_{1}S_{2}A_{T_{j}}^{†};U_{T_{j}}|A_{T_{j}}^{⊃}\right)-\min_{l^{\prime }\in T_{j}\cap \left[1:l\right]}I\left(U_{T_{j}};A_{T_{j},l^{\prime }}^{‡}Y_{l^{\prime }}|A_{T_{j}}^{⊃}\right)$$

*where $A=\{U_{12},U_{1},U_{2}\}$ and the sets $A_{T_{j}}^{-}$, $A_{T_{j}}^{⊃}$, $A_{T_{j}}^{+}$, $A_{T_{j}}^{†}$, $A_{T_{j},1}^{‡}$, $A_{T_{j},2}^{‡}$, evaluated in this case, are given in Table 1. It is easy to see that the region described by *(35)* can be written more explicitly in this case as*

(37a) $$\begin{array}{cc} R_{0} & \geq I(U_{12};S_{1}S_{2}|Y_{1}) \end{array}$$

(37b) $$\begin{array}{cc} R_{0}+R_{2} & \geq \max\left\{I\left(U_{12};S_{1}S_{2}|Y_{1}\right),I\left(U_{12};S_{1}S_{2}|Y_{2}\right)\right\}+I\left(U_{1};S_{1}S_{2}|Y_{1}U_{12}\right)+I\left(U_{2};S_{1}S_{2}|Y_{2}U_{12}\right)\;. \end{array}$$

Also, setting $U_{12}=\left(U_{0},S_{2}\right)$ and $U_{2}=S_{2}$ in *(37)* one recovers the rate-region of Corollary 2. (Such a connection can also be stated for the result of Corollary 1).

### 5.2. Binary Example

**Figure 7:**
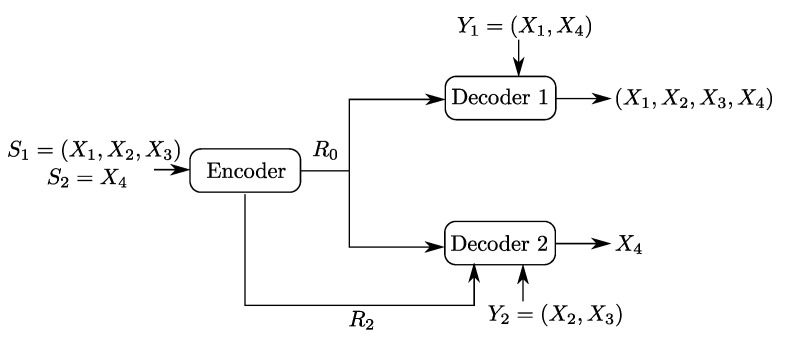
Binary Heegard–Berger example with scalable coding.

Consider the setting of Figure 7. Let $X_{1}$, $X_{2}$, $X_{3}$ and $X_{4}$ be four independent Ber(1/2) random variables. Let the sources be $S_{1}≜\left(X_{1},X_{2},X_{3}\right)$ and $S_{2}≜X_{4}$. Now, consider the Heegard–Berger model with scalable coding shown in Figure 7. The first user, which gets both only the common link, observes the side information $Y_{1}=\left(X_{1},X_{4}\right)$ and wants to reproduce the pair $\left(S_{1},S_{2}\right)$ losslessly. The second user gets both the common and private links, has side information $Y_{2}=\left(X_{2},X_{3}\right)$ and wants to reproduce only the component $S_{2}$, losslessly.

**Claim** **2.** The rate region of the binary Heegard–Berger example with scalable coding of Figure 7 is given by the set of all rate pairs $\left(R_{0},R_{2}\right)$ that satisfy $R_{2}\geq 0$ and $R_{0}\geq 2$.

**Proof.**  The proof of Claim 2 follows easily by specializing, and computing, the result of Remark 9 for the example at hand. First note that

(38a)R0+R2≥H(S2S1|Y2)+minPU0|S1S2H(S1|U0S2Y1)−H(S1|U0S2Y2)(38b)=2+minPU0|S1S2[H(X2X3|X1X4U0)−H(X1|X2X3X4U0)](38c)≥2+minPU0|S1S2[−H(X1|X2U0)](38d)≥1

where equality in all previous inequalities is satisfied with $U_{0}=\left(X_{2},X_{3}\right)$ or with $U_{0}=X_{2}$ or $U_{0}=X_{3}$.

Note as well that the single rate constraint on $R_{0}$ writes as:

(39a)R0≥H(S1S2|Y1)(39b)=2

which renders the sum-rate constraint redundant and ends the proof of the claim. ☐

**Figure 8:**
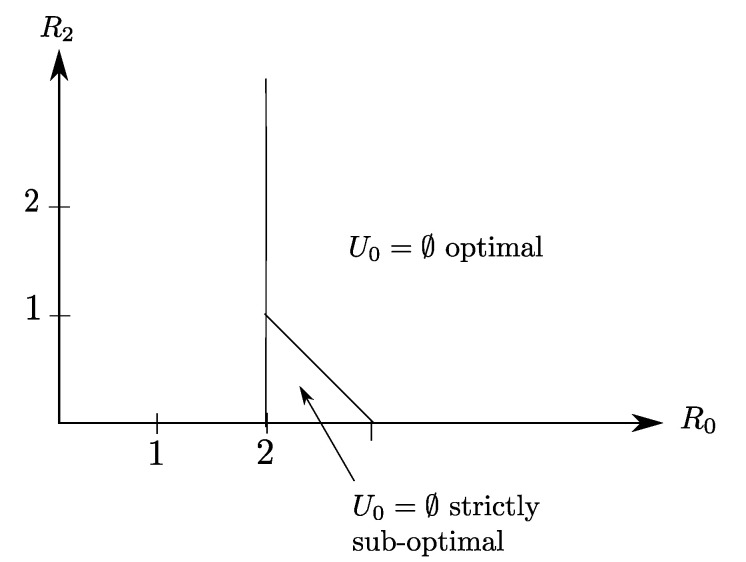
The optimal rate region for the setting of Figure 7 given by ($R_{0}\geq 2,R_{2}\geq 0$). The choice of $U_{0}=⌀$ is optimal only in a slice of the region.

The optimal rate region of Claim 2 is depicted in Figure 8, as the region delimited by the lines $R_{0}=1$ and $R_{2}=0$. Note that for this example, the source component $X_{2}$, which is the only source component that is required by Receiver 2, needs to be transmitted entirely on the common link to also be recovered losslessly by Receiver 1. For this reason, the refinement link is not-constrained and appears to be useless for this example.

There is a sharp difference with the binary Heegard–Berger example with successive refinement of Figure 5 for which the refinement link may sometimes be instrumental to reducing the required rate on the common link. With scalable coding, the refinement link with rate $R_{0}$ does not improve the rate transmitted on the common link.

Also, it is insightful to notice that for this example, because of the side information configuration, the choice $U_{0}=⌀$ in Corollary 2 is strictly suboptimal and results in the smaller region that is described by

(40a) $$\begin{array}{c} R_{0}\geq 2 \end{array}$$

(40b) $$\begin{array}{c} R_{0}+R_{2}\geq 3. \end{array}$$

## 6. Proof of Theorem 1

In the following, we give the proof of the converse part and the direct part of Theorem 1.

The converse part is strongly dependent on the system model we investigate and consists in a series of careful bounding steps resorting to Fano’s inequality, Markov chains and Csiszár–Körner sum-identity.

The proof of achievability is two-fold, and consists in proving a general result that holds for a Gray–Wyner setting with side information, and then deriving the optimal choice of the auxiliary codewords involved for the specific setting with degraded reconstruction sets.

### 6.1. Proof of Converse Part

Assume that a rate triple $\left(R_{0},R_{1},R_{2}\right)$ is $D_{1}$-achievable. Then, let $W_{j}=f_{j}\left(S_{1}^{n},S_{2}^{n}\right)$, where j∈{0,1,2}, be the encoded indices and let ${\hat{S}}_{1}^{n}=g_{1}\left(W_{0},W_{1},Y_{1}^{n}\right)$ be the reconstruction sequence at the first decoder such that $Ed_{1}^{\left(n\right)}\left(S_{1}^{n},{\hat{S}}_{1}^{n}\right)\leq D_{1}$.

Using Fano’s inequality, the lossless reconstruction of the source $S_{2}^{n}$ at both decoders implies that there exists a sequence $\epsilon_{n}\underset{n\to \infty }{\to }0$ such that:

(41) $$\begin{array}{ccc} H(S_{2}^{n}|W_{0}W_{1}Y_{1}^{n}) & \leq & n\epsilon_{n}\;, \end{array}$$

(42) $$\begin{array}{ccc} H(S_{2}^{n}|W_{0}W_{2}Y_{2}^{n}) & \leq & n\epsilon_{n}\;. \end{array}$$

We start by showing the following sum-rate constraint,

(43) $$R_{0}+R_{1}+R_{2}\geq H\left(S_{2}|Y_{2}\right)+I\left(U_{0};S_{1}|S_{2}Y_{2}\right)+I\left(U_{1};S_{1}|U_{0}S_{2}Y_{1}\right)\;.$$

We have that

n(R0+R1+R2)(44a)≥H(W0)+H(W2)+H(W1)(44b)≥H(W0)+H(W2|W0)+H(W1)(44c)=H(W0W2)+H(W1)(44d)≥H(W0W2|Y2n)+H(W1|W0S2nY1n)(44e)≥I(W0W2;S1nS2n|Y2n)+I(W1;S1n|W0S2nY1n)(44f)=H(S1nS2n|Y2n)−H(S1nS2n|W0W2Y2n)+H(S1n|W0S2nY1n)−H(S1n|W0W1S2nY1n)(44g)≥(a)H(S1nS2n|Y2n)−H(S1n|W0W2S2nY2n)+H(S1n|W0S2nY1n)−H(S1n|W0W1S2nY1n)−nϵn(44h)≥H(S1nS2n|Y2n)−H(S1n|W0S2nY2n)+H(S1n|W0S2nY1n)−H(S1n|W0W1S2nY1n)−nϵn

where (a) in (44) stems from Fano’s inequality (42), which results from the lossless reconstruction of $S_{2}^{n}$ at receiver 2.

Let us define then:

(45) $$\begin{array}{ccc} A & ≜ & H\left(S_{1}^{n}|W_{0}S_{2}^{n}Y_{1}^{n}\right)-H\left(S_{1}^{n}|W_{0}S_{2}^{n}Y_{2}^{n}\right)\;, \end{array}$$

(46) $$\begin{array}{ccc} B & ≜ & H(S_{1}^{n}|W_{0}W_{1}S_{2}^{n}Y_{1}^{n})\;. \end{array}$$

In the following, we aim for single-letter bounds on the two quantities *A* and *B*.

Since the side information sequences $Y_{1}^{n}$ and $Y_{2}^{n}$ are not degraded and do not exhibit any structure, together with the sources $\left(S_{1}^{n},S_{2}^{n}\right)$, single-letterizing the quantity A can be obtained through some judicious bounding steps that are reported below, in which some important Markov chain are shown to hold and quantities are manipulated appropriately, together with several invocations of Csiszár–Körner sum identity .

Let us start by writing that

(47a)A≜H(S1n|W0S2nY1n)−H(S1n|W0S2nY2n)(47b)=I(S1n;Y2n|W0S2n)−I(S1n;Y1n|W0S2n)(47c)=∑i=1nI(S1n;Y2,i|W0Y2i−1S2n)−I(S1n;Y1,i|W0Y1,i+1nS2n)(47d)=(a)∑i=1nI(S1nY1,i+1n;Y2,i|W0Y2i−1S2n)−I(S1nY2i−1;Y1,i|W0Y1,i+1nS2n)(47e)=(b)∑i=1nI(S1n;Y2,i|W0Y2i−1Y1,i+1nS2n)−I(S1n;Y1,i|W0Y2i−1Y1,i+1nS2n)(47f)=(c)∑i=1nI(S1,i;Y2,i|W0Y2i−1Y1,i+1nS2n)−I(S1,i;Y1,i|W0Y2i−1Y1,i+1nS2n)(47g)=∑i=1nH(S1,i|Y1,iW0Y2i−1Y1,i+1nS2n)−H(S1,i|Y2,iW0Y2i−1Y1,i+1nS2n)(47h)=∑i=1nH(S1,i|Y1,iS2,iU0,i)−H(S1,i|Y2,iS2,iU0,i)

where $U_{0,i}≜\left(W_{0},Y_{2}^{i-1},Y_{1,i+1}^{n},S_{2,<i>}\right)$ (note that the lossless reconstruction of $S_{2}^{n}$ at both receivers is instrumental to the definition of $U_{0}$ which plays the role of the common auxiliary variable in the proof of converse), and where (a) in (47) follows using the following Csiszár–Körner sum-identity

(48) $$\sum_{i=1}^{n}I\left(Y_{2}^{i-1};Y_{1,i}|S_{1}^{n}W_{0}Y_{1,i+1}^{n}S_{2}^{n}\right)=\sum_{i=1}^{n}I\left(Y_{1,i+1}^{n};Y_{2,i}|S_{1}^{n}W_{0}Y_{2}^{i-1}S_{2}^{n}\right),$$

(b) in (47) follows using the Csiszár–Körner sum-identity given by

(49) $$\begin{array}{c} \sum_{i=1}^{n}I\left(Y_{2}^{i-1};Y_{1,i}|W_{0}Y_{1,i+1}^{n}S_{2}^{n}\right)=\sum_{i=1}^{n}I\left(Y_{1,i+1}^{n};Y_{2,i}|W_{0}Y_{2}^{i-1}S_{2}^{n}\right)\;, \end{array}$$

while (c) in (47) is the consequence of the following sequence of Markov chains

(50a) (S1i−1,S1,i+1n,S2i−1,S2,i+1n,Y1,i+1n,Y2i−1)○(S1,i,S2,i)○Yj,i

(50b) ⇒(a)(S1i−1,S1,i+1n,S2i−1,S2,i+1n,Y1,i+1n,Y2i−1,W0)○(S1,i,S2,i)○Yj,i

(50c) ⇒(S1i−1,S1,i+1n)○(S2i−1,S2,i+1n,Y1,i+1n,Y2i−1,W0,S1,i,S2,i)○Yj,i

where (50a) results from that the source sequences $\left(S_{1}^{n},S_{2}^{n},Y_{1}^{n},Y_{2}^{n}\right)$ are memoryless, while (a) in (50) is a consequence of that $W_{0}$ is a function of the pair of sequences $\left(S_{1}^{n},S_{2}^{n}\right)$.

To upper-bound the term B, note the following

(51a)B≜H(S1n|W0W1S2nY1n)(51b)=∑i=1nH(S1,i|W0W1S2nY1nS1i−1)(51c)=∑i=1nH(S1,i|S2,iY1,iW0S2,<i>Y1,i+1nS1i−1W1Y1i−1)(51d)=(a)∑i=1nH(S1,i|S2,iY1,iW0S2,<i>Y1,i+1nS1i−1Y2i−1W1Y1i−1)(51e)≤∑i=1nH(S1,i|S2,iY1,iW0S2,<i>Y1,i+1nY2i−1W1Y1i−1)

where (a) in (51) is a consequence of the following sequence of Markov chains:

(52a)Y2i−1○(S1i−1,S2i−1,Y1i−1)○(S1,i,S1,i+1n,S2,i,S2,i+1n,Y1,i+1n)(52b)⇒(a)Y2i−1○(S1i−1,S2i−1,Y1i−1)○(S1,i,S1,i+1n,S2,i,S2,i+1n,Y1,i+1n,W0,W1)(52c)⇒Y2i−1○(S1i−1,S2i−1,Y1i−1,S2,i,S2i−1,Y1,i+1n,W0,W1)○S1,i.

where (52a) results from that the source sequences $\left(S_{1}^{n},S_{2}^{n},Y_{1}^{n},Y_{2}^{n}\right)$ are memoryless, while (a) in (52) is a consequence of that $W_{0}$ and $W_{1}$ are each function of the pair of sequences $\left(S_{1}^{n},S_{2}^{n}\right)$.

Finally, letting $U_{1,i}≜\left(W_{1},Y_{1}^{i-1}\right)$ so that the choice of $\left(U_{0,i},U_{1,i}\right)$ satisfy the condition ${\hat{S}}_{1,i}=g_{i}\left(Y_{1,i},U_{0,i},U_{1,i},S_{2,i}\right)$, we write the resulting sum-rate constraint as

(53) $$\begin{array}{ccc} n(R_{0}+R_{1}+R_{2}) & \geq & nH\left(S_{1}S_{2}|Y_{2}\right)+\sum_{i=1}^{n}\left[H\left(S_{1,i}|S_{2,i}Y_{1,i}U_{0,i}\right)-H\left(S_{1,i}|S_{2,i}Y_{2,i}U_{0,i}\right)\right]\\ & & \;\;-\sum_{i=1}^{n}H\left(S_{1,i}|S_{2,i}Y_{1,i}U_{0,i}U_{1,i}\right)-n\epsilon_{n}. \end{array}$$

Let us now prove that the following bound holds

(54) $$R_{0}+R_{1}\geq H\left(S_{2}S_{1}|Y_{1}\right)-H\left(S_{1}|U_{0}U_{1}Y_{1}S_{2}\right)\;.$$

We have

(55a)n(R0+R1)≥H(W0)+H(W1|W0)(55b)=H(W0,W1)(55c)≥H(W0W1|Y1n)(55d)≥I(W0W1;S1nS2n|Y1n)(55e)=H(S1nS2n|Y1n)−H(S1nS2n|W0W1Y1n)(55f)≥(a)H(S1nS2n|Y1n)−H(S1n|W0W1S2nY1n)−nϵn(55g)=nH(S1S2|Y1)−B−nϵn(55h)≥(b)nH(S1S2|Y1)−∑i=1nH(S1,i|S2,iY1,iU0,iU1,i)−nϵn,

where (a) in (55) is a consequence of Fano’s inequality in (41), which results from the lossless reconstruction of $S_{2}^{n}$ at receiver 1, and (b) in (55) results from the upper bound on B in (51e). As for the third rate constraint

(56) $$R_{0}+R_{2}\geq H\left(S_{1}S_{2}|Y_{2}\right)-H\left(S_{1}|U_{0}Y_{2}S_{2}\right)\;,$$

we write

(57a)n(R0+R2)≥H(W0W2)(57b)≥H(W0W2|Y2n)(57c)≥I(W0W2;S1nS2n|Y2n)(57d)=H(S1nS2n|Y2n)−H(S1nS2n|W0W2Y2n)(57e)≥(a)H(S1nS2n|Y2n)−H(S1n|W0W2S2nY2n)−nϵn(57f)≥H(S1nS2n|Y2n)−H(S1n|W0S2nY2n)−nϵn(57g)=nH(S1S2|Y2)−∑i=1nH(S1,i|S2,iY2,iW0S2,<i>Y2,<i>S1,i+1n)−nϵn(57h)=(b)nH(S1S2|Y2)−∑i=1nH(S1,i|S2,iY2,iW0S2,<i>Y2,<i>S1,i+1nY1,i+1n)−nϵn(57i)≥nH(S1S2|Y2)−∑i=1nH(S1,i|S2,iY2,iW0S2,<i>Y2i−1Y1,i+1n)−nϵn(57j)=nH(S1S2|Y2)−∑i=1nH(S1,i|S2,iY2,iU0,i)−nϵn.

where (a) in (57) is a consequence of Fano’s inequality in (42) and (b) in (57) stems for the following sequence of Markov Chains.

(58a)Y1,i+1n○(S2,i+1n,S1,i+1n,Y1,i+1n)○(S1,i,S1i−1,S2,i,S2i−1,Y1i−1)(58b)⇒(a)Y1,i+1n○(S2,i+1n,S1,i+1n,Y1,i+1n)○(S1,i,S1i−1,S2,i,S2i−1,Y1i−1,W0,W1)(58c)⇒Y1,i+1n○(S2,i+1n,S1,i+1n,Y1,i+1n,S2,i,S2i−1,Y1i−1,W0,W1)○S1,i.

where (58a) results from that the source sequences $\left(S_{1}^{n},S_{2}^{n},Y_{1}^{n},Y_{2}^{n}\right)$ are memoryless, while (a) in (58) is a consequence of that $W_{0}$ and $W_{1}$ are each function of the pair of sequences $\left(S_{1}^{n},S_{2}^{n}\right)$.

Let *Q* be an integer-valued random variable, ranging from 1 to *n*, uniformly distributed over [1:*n*] and independent of all other variables $\left(S_{1},S_{2},U_{0},U_{1},Y_{1},Y_{2}\right)$. We have

R0+R1+R2≥H(S1S2|Y2)+1n∑i=1nH(S1,i|S2,iY1,iU0,i)−H(S1,i|S2,iY2,iU0,i)(59a)−1n∑i=1nH(S1,i|S2,iY1,iU0,iU1,i)−nϵn=H(S1S2|Y2)+∑i=1nP(Q=i)H(S1,Q|S2,QY1,QU0,Q,Q=i)−H(S1,Q|S2,QY2,QU0,Q,Q=i)(59b)−∑i=1nP(Q=i)H(S1,Q|S2,QY1,QU0,QU1,Q,Q=i)−nϵn=H(S1S2|Y2)+H(S1,Q|S2,QY1,QU0,QQ)−H(S1,Q|S2,QY2,QU0,QQ)(59c)−H(S1,Q|S2,QY1,QU0,QU1,QQ)−nϵn=(a)H(S1S2|Y2)+H(S1|S2Y1U0,QQ)−H(S1|S2Y2U0,QQ)(59d)−H(S1|S2Y1U0,QU1,QQ)−nϵn

where (a) in (59) is a consequence of that all sources $\left(S_{1}^{n},S_{2}^{n},Y_{1}^{n},Y_{2}^{n}\right)$ are memoryless.

Let us now define $U_{1}≜\left(Q,U_{1,Q}\right)$ and $U_{0}≜\left(Q,U_{0,Q}\right)$, we obtain

(60) $$R_{0}+R_{1}+R_{2}\geq H\left(S_{1}S_{2}|Y_{2}\right)+H\left(S_{1}|S_{2}Y_{1}U_{0}\right)-H\left(S_{1}|S_{2}Y_{2}U_{0}\right)-H\left(S_{1}|S_{2}Y_{1}U_{0}U_{1}\right)\;.$$

The two other rate constraints can be written in a similar fashion,

(61a) $$\begin{array}{ccc} R_{0}+R_{1} & \geq & H\left(S_{2}S_{1}|Y_{1}\right)-H\left(S_{1}|U_{0}U_{1}Y_{1}S_{2}\right) \end{array}$$

(61b) $$\begin{array}{ccc} R_{0}+R_{2} & \geq & H\left(S_{1}S_{2}|Y_{2}\right)-H\left(S_{1}|U_{0}Y_{2}S_{2}\right)\;; \end{array}$$

and this completes the proof of converse.  ☐

### 6.2. Proof of Direct Part

We first show that the rate-distortion region of the proposition that will follow is achievable. The achievability of the rate-distortion region of Theorem 1 follows by choosing then the random variable $V_{0}$ of the proposition as $V_{0}=\left(U_{0},S_{2}\right)$.

**Proposition** **1.** *An inner bound on the rate-distortion region of the Gray–Wyner model with side information and degraded reconstruction sets of Figure 2 is given by the set of all rate-distortion quadruples $\left(R_{0},R_{1},R_{2},D_{1}\right)$ that satisfy*

(62a) $$\begin{array}{cc} R_{0}+R_{1} & \geq I(V_{0}U_{1};S_{1}S_{2}|Y_{1}) \end{array}$$

(62b) $$\begin{array}{cc} R_{0}+R_{2} & \geq I(V_{0};S_{1}S_{2}|Y_{2}) \end{array}$$

(62c) $$\begin{array}{cc} R_{0}+R_{1}+R_{2} & \geq \max\;\left\{I\left(V_{0};S_{1}S_{2}|Y_{1}\right),I\left(V_{0};S_{1}S_{2}|Y_{2}\right)\right\}+I\left(U_{1};S_{1}S_{2}|V_{0}Y_{1}\right) \end{array}$$

*for some choice of the random variables $\left(V_{0},U_{1}\right)$ such that (V0,U1)○(S1,S2)○(Y1,Y2) and there exist functions $g_{1}$, $g_{2,1}$, and $g_{2,2}$ such that:*

(63a) $$\begin{array}{cc} {\hat{S}}_{1} & =g_{1}\left(V_{0},U_{1},Y_{1}\right) \end{array}$$

(63b) $$\begin{array}{cc} S_{2} & =g_{2,1}\left(V_{0},U_{1},Y_{1}\right) \end{array}$$

(63c) $$\begin{array}{cc} S_{2} & =g_{2,2}\left(V_{0},Y_{2}\right)\;, \end{array}$$

*and*

(64) $$Ed_{1}\left(S_{1};{\hat{S}}_{1}\right)\leq D_{1}.$$

**Proof** **of** **Proposition** **1.** We now describe a coding scheme that achieves the rate-distortion region of Proposition 1. The scheme is very similar to one that is developed by Shayevitz and Wigger (Theorem 2, [4]) for a Gray–Wyner model with side information. In particular, similar to (Theorem 2, [4]), it uses a double-binning technique for the common codebook, one that is relevant for Receiver 1 and one that is relevant for Receiver 2. Note, however, that, formally, the result of Proposition 1 cannot be obtained by readily applying (Theorem 2, [4]) as is; and one needs to extend the result of (Theorem 2, [4]) in a manner that accounts for that the source component $S_{2}^{n}$ is to be recovered losslessly by both decoders. This can be obtained by extending the distortion measure of (Theorem 2, [4]) to one that is vector-valued, i.e., $d\left(\left(s_{1},s_{2}\right),\left({\hat{s}}_{1},{\hat{s}}_{2}\right)\right)=\left(d_{1}\left(s_{1},{\hat{s}}_{1}\right),d_{H}\left(s_{2},{\hat{s}}_{2}\right)\right)$, where $d_{H}\left(\cdot ,\cdot \right)$ denotes the Hamming distance. For reasons of completeness, we provide here a proof of Proposition 1.

Our scheme has the following parameters: a conditional joint pmf $P_{V_{0}U_{1}|S_{1}S_{2}}$ that satisfies (63) and (64), and non-negative communication rates $T_{0}$, $T_{1}$, $T_{0,0}$, $T_{0,p}$, $T_{1,0}$, $T_{1,1}$, ${\tilde{R}}_{0,0}$, ${\tilde{R}}_{0,1}$, ${\tilde{R}}_{0,2}$, ${\tilde{R}}_{1,0}$ and ${\tilde{R}}_{1,1}$ such that

(65a) $$\begin{array}{cc} T_{0} & =T_{0,0}+T_{0,p}\;,\;0\leq {\tilde{R}}_{0,0}\leq T_{0,0}\;,\;0\leq {\tilde{R}}_{0,1}\leq T_{0,p}\;,\;0\leq {\tilde{R}}_{0,2}\leq T_{0,p} \end{array}$$

(65b) $$\begin{array}{cc} T_{1} & =T_{1,0}+T_{1,1}\;,\;0\leq {\tilde{R}}_{1,0}\leq T_{1,0}\;,\;0\leq {\tilde{R}}_{1,1}\leq T_{1,1}. \end{array}$$

#### 6.2.1. Codebook Generation

(1) Randomly and independently generate $2^{nT_{0}}$ length-*n* codewords $v_{0}^{n}\left(k_{0}\right)$ indexed with the pair of indices $k_{0}=\left(k_{0,0},k_{0,p}\right)$, where $k_{0,0}\in \left[1:2^{nT_{0,0}}\right]$ and $k_{0,p}\in \left[1:2^{nT_{0,p}}\right]$. Each codeword $v_{0}^{n}\left(k_{0}\right)$ has i.i.d. entries drawn according to $\prod_{i=1}^{n}P_{V_{0}}\left(v_{0,i}\left(k_{0}\right)\right)$. The codewords $\left\{v_{0}^{n}\left(k_{0}\right)\right\}$ are partitioned into superbins whose indices will be relevant for both receivers; and each superbin is partitioned int two different ways, each into subbins whose indices will be relevant for a distinct receiver (i.e., double-binning). This is obtained by partitioning the indices $\left\{\left(k_{0,0},k_{0,p}\right)\right\}$ as follows. We partition the $2^{nT_{0,0}}$ indices $\left\{k_{0,0}\right\}$ into $2^{n{\tilde{R}}_{0,0}}$ bins by randomly and independently assigning each index $k_{0,0}$ to an index ${\tilde{w}}_{0,0}\left(k_{0,0}\right)$ according to a uniform pmf over $\left[1:2^{n{\tilde{R}}_{0,0}}\right]$. We refer to each subset of indices $\left\{k_{0,0}\right\}$ with the same index ${\tilde{w}}_{0,0}$ as a bin $B_{00}\left({\tilde{w}}_{0,0}\right)$, ${\tilde{w}}_{0,0}\in \left[1:2^{n{\tilde{R}}_{0,0}}\right]$. In addition, we make two distinct partitions of the $2^{nT_{0,p}}$ indices $\left\{k_{0,p}\right\}$, each relevant for a distinct receiver. In the first partition, which is relevant for Receiver 1, the indices $\left\{k_{0,p}\right\}$ are assigned randomly and independently each to an index ${\tilde{w}}_{0,1}\left(k_{0,p}\right)$ according to a uniform pmf over $\left[1:2^{n{\tilde{R}}_{0,1}}\right]$. We refer to each subset of indices $\left\{k_{0,p}\right\}$ with the same index ${\tilde{w}}_{0,1}$ as a bin $B_{01}\left({\tilde{w}}_{0,1}\right)$, ${\tilde{w}}_{0,1}\in \left[1:2^{n{\tilde{R}}_{0,1}}\right]$. Similarly, in the second partition, which is relevant for Receiver 2, the indices $\left\{k_{0,p}\right\}$ are assigned randomly and independently each to an index ${\tilde{w}}_{0,2}\left(k_{0,p}\right)$ according to a uniform pmf over $\left[1:2^{n{\tilde{R}}_{0,2}}\right]$; and refer to each subset of indices $\left\{k_{0,p}\right\}$ with the same index ${\tilde{w}}_{0,2}$ as a bin $B_{02}\left({\tilde{w}}_{0,2}\right)$, ${\tilde{w}}_{0,2}\in \left[1:2^{n{\tilde{R}}_{0,2}}\right]$.
(2) For each $k_{0}\in \left[1:2^{nT_{0}}\right]$, randomly and independently generate $2^{nT_{1}}$ length-*n* codewords $u_{1}^{n}\left(k_{1},k_{0}\right)$ indexed with the pair of indices $k_{1}=\left(k_{1,0},k_{1,1}\right)$, where $k_{1,0}\in \left[1:2^{nT_{1,0}}\right]$ and $k_{1,1}\in \left[1:2^{nT_{1,1}}\right]$. Each codeword $u_{1}^{n}\left(k_{1},k_{0}\right)$ is with i.i.d. elements drawn according to $\prod_{i=1}^{n}P_{U_{1}|V_{0}}\left(u_{1,i}\left(k_{1},k_{0}\right)|v_{0,i}\left(k_{0}\right)\right)$. We partition the $2^{nT_{1,0}}$ indices $\left\{k_{1,0}\right\}$ into $2^{n{\tilde{R}}_{1,0}}$ bins by randomly and independently assigning each index $k_{1,0}$ to an index ${\tilde{w}}_{1,0}\left(k_{1,0}\right)$ according to a uniform pmf over $\left[1:2^{n{\tilde{R}}_{1,0}}\right]$. We refer to each subset of indices $\left\{k_{1,0}\right\}$ with the same index ${\tilde{w}}_{1,0}$ as a bin $B_{10}\left({\tilde{w}}_{1,0}\right)$, ${\tilde{w}}_{1,0}\in \left[1:2^{n{\tilde{R}}_{1,0}}\right]$. Similarly, we partition the $2^{nT_{1,1}}$ indices $\left\{k_{1,1}\right\}$ into $2^{n{\tilde{R}}_{1,1}}$ bins by randomly and independently assigning each index $k_{1,1}$ to an index ${\tilde{w}}_{1,1}\left(k_{1,1}\right)$ according to a uniform pmf over $\left[1:2^{n{\tilde{R}}_{1,1}}\right]$; and refer to each subset of indices $\left\{k_{1,1}\right\}$ with the same index ${\tilde{w}}_{1,1}$ as a bin $B_{11}\left({\tilde{w}}_{1,1}\right)$, ${\tilde{w}}_{1,1}\in \left[1:2^{n{\tilde{R}}_{1,1}}\right]$.
(3) Reveal all codebooks and their partitions to the encoder, the codebook of $\left\{v_{0}^{n}\left(k_{0}\right)\right\}$ and its partitions to both receivers, and the codebook of $\left\{u_{1}^{n}\left(k_{1},k_{0}\right)\right\}$ and its partitions to only Receiver 1.

#### 6.2.2 Encoding

Upon observing the source pair $\left(S_{1}^{n},S_{2}^{n}\right)=\left(s_{1}^{n},s_{2}^{n}\right)$, the encoder finds an index $k_{0}=\left(k_{0,0},k_{0,p}\right)$ such that the codeword $v_{0}^{n}\left(k_{0}\right)$ is jointly typical with $\left(s_{1}^{n},s_{2}^{n}\right)$, i.e.,

(66) $$\left(s_{1}^{n},s_{2}^{n},v_{0}^{n}\left(k_{0}\right)\right)\in T_{\left[S_{1}S_{2}V_{0}\right]}^{\left(n\right)}\;.$$

By the covering lemma (Chapter 3, [16]), the encoding in this step is successful as long as *n* is large and

(67) $$T_{0}\geq I\left(V_{0};S_{1}S_{2}\right).$$

Next, it finds an index $k_{1}=\left(k_{1,0},k_{1,1}\right)$ such that the codeword $u_{1}^{n}\left(k_{1},k_{0}\right)$ is jointly typical with the triple $\left(s_{1}^{n},s_{2}^{n},v_{0}^{n}\left(k_{0}\right)\right)$, i.e.,

(68) $$\left(s_{1}^{n},s_{2}^{n},v_{0}^{n}\left(k_{0}\right),u_{1}^{n}\left(k_{1},k_{0}\right)\right)\in T_{\left[S_{1}S_{2}V_{0}U_{1}\right]}^{\left(n\right)}.$$

Again, by the covering lemma (Chapter 3, [16]), the encoding in this step is successful as long as *n* is large and

(69) $$T_{1}\geq I\left(U_{1};S_{1}S_{2}|V_{0}\right).$$

Let ${\tilde{w}}_{0,0}$, ${\tilde{w}}_{0,1}$ and ${\tilde{w}}_{0,2}$ be the bin indices such that $k_{0,0}\in B_{00}\left({\tilde{w}}_{0,0}\right)$, $k_{0,p}\in B_{01}\left({\tilde{w}}_{0,1}\right)$ and $k_{0,p}\in B_{02}\left({\tilde{w}}_{0,2}\right)$. In addition, let ${\tilde{w}}_{1,0}$ and ${\tilde{w}}_{1,1}$ be the bin indices such that $k_{1,0}\in B_{10}\left({\tilde{w}}_{1,0}\right)$ and $k_{1,1}\in B_{11}\left({\tilde{w}}_{1,1}\right)$. The encoder then sends the product message $W_{0}=\left({\tilde{w}}_{0,0},{\tilde{w}}_{1,0}\right)$ over the error-free rate-limited common link of capacity $R_{0}$. In addition, it sends the product message $W_{1}=\left({\tilde{w}}_{0,1},{\tilde{w}}_{1,1}\right)$ over the error-free rate-limited individual link to Receiver 1 of capacity $R_{1}$, and the message $W_{2}={\tilde{w}}_{0,2}$ over the error-free rate-limited individual link to Receiver 2 of capacity $R_{2}$.

#### 6.2.3 Decoding

Receiver 1 gets the messages $\left(W_{0},W_{1}\right)=\left({\tilde{w}}_{0,0},{\tilde{w}}_{1,0},{\tilde{w}}_{0,1},{\tilde{w}}_{1,1}\right)$. It seeks a codeword $v_{0}^{n}\left(k_{0}\right)$ and a codeword $u_{1}^{n}\left(k_{1},k_{0}\right)$, with the indices $k_{0}=\left(k_{0,0},k_{0,p}\right)$ and $k_{1}=\left(k_{1,0},k_{1,1}\right)$ satisfying $k_{0,0}\in B_{00}\left({\tilde{w}}_{0,0}\right)$, $k_{0,p}\in B_{01}\left({\tilde{w}}_{0,1}\right)$, $k_{1,0}\in B_{10}\left({\tilde{w}}_{1,0}\right)$ and $k_{1,1}\in B_{11}\left({\tilde{w}}_{1,1}\right)$, and such that

(70) $$\left(v_{0}^{n}\left(k_{0}\right),u_{1}^{n}\left(k_{1},k_{0}\right),y_{1}^{n}\right)\in T_{\left[V_{0}U_{1}Y_{1}\right]}^{\left(n\right)}\;.$$

By the multivariate packing lemma (Chapter 12, [16]), the error in this decoding step at Receiver 1 vanishes exponentially as long as *n* is large and

(71a) $$\begin{array}{cc} T_{0,0}-{\tilde{R}}_{0,0}+T_{0,p}-{\tilde{R}}_{0,1} & \leq I(V_{0};Y_{1}) \end{array}$$

(71b) $$\begin{array}{cc} T_{1,0}-{\tilde{R}}_{1,0}+T_{1,1}-{\tilde{R}}_{1,1} & \leq I(U_{1};Y_{1}|V_{0})\;. \end{array}$$

Receiver 1 then sets its reproduced codewords ${\hat{s}}_{2,1}^{n}$ and ${\hat{s}}_{1}^{n}$ , respectively, as

(72a) $$\begin{array}{cc} {\hat{s}}_{2,1}^{n} & =g_{2,1}\left(v_{0}^{n}\left(k_{0}\right),u_{1}^{n}\left(k_{1},k_{0}\right),y_{1}^{n}\right) \end{array}$$

(72b) $$\begin{array}{cc} {\hat{s}}_{1}^{n} & =g_{1}\left(v_{0}^{n}\left(k_{0}\right),u_{1}^{n}\left(k_{1},k_{0}\right),y_{1}^{n}\right)\;. \end{array}$$

Similary, Receiver 2 gets the message $\left(W_{0},W_{2}\right)=\left({\tilde{w}}_{0,0},{\tilde{w}}_{1,0},{\tilde{w}}_{0,2}\right)$. It seeks a codeword $v_{0}^{n}\left(k_{0}\right)$, with $k_{0}=\left(k_{0,0},k_{0,p}\right)$ satisfying $k_{0,0}\in B_{00}\left({\tilde{w}}_{0,0}\right)$ and $k_{0,p}\in B_{02}\left({\tilde{w}}_{0,2}\right)$, and such that

(73) $$\left(v_{0}^{n}\left(k_{0}\right),y_{1}^{n}\right)\in T_{\left[V_{0}Y_{2}\right]}^{\left(n\right)}\;.$$

Again, using the multivariate packing lemma (Chapter 12, [16]), the error in this decoding step at Receiver 2 vanishes exponentially as long as *n* is large and

(74) $$T_{0,0}-{\tilde{R}}_{0,0}+T_{0,p}-{\tilde{R}}_{0,2}\leq I\left(V_{0};Y_{2}\right).$$

Receiver 2 then sets its reconstructed codeword ${\hat{s}}_{2,1}^{n}$ as

(75) $${\hat{s}}_{2,2}^{n}=g_{2,2}\left(v_{0}^{n}\left(k_{0}\right),y_{2}^{n}\right)\;.$$

Summarizing, combining Equations (67), (69), (71) and (74), the communication rates $T_{0}$, $T_{1}$, $T_{0,0}$, $T_{0,p}$, $T_{1,0}$, $T_{1,1}$, ${\tilde{R}}_{0,0}$, ${\tilde{R}}_{0,1}$, ${\tilde{R}}_{0,2}$, ${\tilde{R}}_{1,0}$ and ${\tilde{R}}_{1,1}$ satisfy the following inequalities

(76a) $$\begin{array}{cc} T_{0} & \geq I(V_{0};S_{1}S_{2}) \end{array}$$

(76b) $$\begin{array}{cc} T_{1} & \geq I(U_{1};S_{1}S_{2}|V_{0}) \end{array}$$

(76c) $$\begin{array}{cc} T_{0,0}-{\tilde{R}}_{0,0}+T_{0,p}-{\tilde{R}}_{0,1} & \leq I(V_{0};Y_{1}) \end{array}$$

(76d) $$\begin{array}{cc} T_{0,0}-{\tilde{R}}_{0,0}+T_{0,p}-{\tilde{R}}_{0,2} & \leq I(V_{0};Y_{2}) \end{array}$$

(76e) $$\begin{array}{cc} T_{1,0}-{\tilde{R}}_{1,0}+T_{1,1}-{\tilde{R}}_{1,1} & \leq I(U_{1};Y_{1}|V_{0}). \end{array}$$

Choosing ${\tilde{R}}_{0,0}$, ${\tilde{R}}_{1,1}$, ${\tilde{R}}_{0,2}$, ${\tilde{R}}_{1,0}$ and ${\tilde{R}}_{1,1}$ to also satisfy the rate relations

(77a) $$\begin{array}{cc} R_{0} & ={\tilde{R}}_{0,0}+{\tilde{R}}_{1,0} \end{array}$$

(77b) $$\begin{array}{cc} R_{1} & ={\tilde{R}}_{0,1}+{\tilde{R}}_{1,1} \end{array}$$

(77c) $$\begin{array}{cc} R_{2} & ={\tilde{R}}_{0,2}. \end{array}$$

and, finally, using Fourier-Motzkin elimination (FME) to successively project out the nuisance variables $T_{0,0}$, $T_{0,p}$, $T_{1,0}$, $T_{1,1}$, $T_{0}$, $T_{1}$, and then ${\tilde{R}}_{0,0}$, ${\tilde{R}}_{0,1}$, ${\tilde{R}}_{0,2}$, ${\tilde{R}}_{1,0}$ and ${\tilde{R}}_{1,1}$ from the set of relations formed by (65), (76) and (77), we get the region of Proposition 1.

This completes the proof of the proposition; and so that of the direct part of Theorem 1. ☐

## Figures and Tables

**Table 1:** Auxiliary random variables associated with the subsets that appear in (36).

	$T_{0}$	$T_{1}$	$T_{2}$
$A_{T_{j}}^{-}$	∅	∅	$U_{1}$
$A_{T_{j}}^{⊃}$	∅	$U_{12}$	$U_{12}$
$A_{T_{j}}^{+}$	$\left\{U_{1},U_{2}\right\}$	∅	∅
$A_{T_{j}}^{†}$	∅	∅	∅
$A_{T_{j},1}^{‡}$	∅	∅	∅
$A_{T_{j},2}^{‡}$	∅	∅	∅
